# Allele exchange at the EPSPS locus confers glyphosate tolerance in cassava

**DOI:** 10.1111/pbi.12868

**Published:** 2018-01-22

**Authors:** Aaron W. Hummel, Raj Deepika Chauhan, Tomas Cermak, Andrew M. Mutka, Anupama Vijayaraghavan, Adam Boyher, Colby G. Starker, Rebecca Bart, Daniel F. Voytas, Nigel J. Taylor

**Affiliations:** ^1^ Department of Genetics, Cell Biology, & Development and Center for Genome Engineering University of Minnesota Minneapolis MN USA; ^2^ Donald Danforth Plant Science Center St. Louis MO USA; ^3^ Present address: KWS Gateway Research Center St. Louis MO USA; ^4^ Present address: Inari Agriculture Inc 200 Sidney St Suite 340, Cambridge MA 02139 USA; ^5^ Present address: Elemental Enzymes St. Louis MO USA; ^6^ Present address: Monsanto St. Louis MO USA

**Keywords:** gene replacement, gene editing, cassava, herbicide tolerance

## Abstract

Effective weed control can protect yields of cassava (*Manihot esculenta*) storage roots. Farmers could benefit from using herbicide with a tolerant cultivar. We applied traditional transgenesis and gene editing to generate robust glyphosate tolerance in cassava. By comparing promoters regulating expression of transformed 5‐enolpyruvylshikimate‐3‐phosphate synthase (EPSPS) genes with various paired amino acid substitutions, we found that strong constitutive expression is required to achieve glyphosate tolerance during *in vitro* selection and in whole cassava plants. Using strategies that exploit homologous recombination (HR) and nonhomologous end‐joining (NHEJ) DNA repair pathways, we precisely introduced the best‐performing allele into the cassava genome, simultaneously creating a promoter swap and dual amino acid substitutions at the endogenous EPSPS locus. Primary EPSPS‐edited plants were phenotypically normal, tolerant to high doses of glyphosate, with some free of detectable T‐DNA integrations. Our methods demonstrate an editing strategy for creating glyphosate tolerance in crop plants and demonstrate the potential of gene editing for further improvement of cassava.

## Introduction

Among chemical weed control agents, the broad‐spectrum herbicide glyphosate is favoured for its high efficacy, low cost, low environmental persistence, flexible application requirements and safety (Duke and Powles, 2008). Glyphosate competitively inhibits the EPSPS‐catalysed synthesis of aromatic amino acids and secondary metabolites in plant chloroplasts by mimicking a transition state of 5‐enolpyruvylshikimate‐3‐phosphate from phosphoenol pyruvate and shikimate‐3‐phosphate (Schönbrunn *et al*., 2001). Amino acid substitutions that render plant EPSPS less sensitive to glyphosate are well characterized (Sammons and Gaines, 2014), and gene editing approaches have been used to introduce some of these mutations into crop plants. For example, the T102I/P106S (TIPS) and T102I/P106A (TIPA) double amino acid substitutions were introduced into rice (Li *et al*., 2016) (*Oryza sativa*) and flax (Sauer *et al*., 2016) (*Linum usitatissimum*), respectively. However, tests of glyphosate tolerance at the whole plant level were assessed with low concentrations of active ingredient (AI) (<0.5%), leaving uncertainty as to whether this approach can achieve robust tolerance in the field. In commercialized GA21 transgenic corn (Monsanto, 2002), the TIPS enzyme, expressed from the strong constitutive rice actin promoter and intron, was present in 3–5 functional copies, suggesting high expression is necessary to achieve agronomically relevant glyphosate tolerance. TIPS‐edited rice, as well as spontaneous TIPS mutations in wild goosegrass (*Eleusine indica*), reduced fitness in the absence of herbicide, presumably due to the lower catalytic efficiency of the mutated enzyme (Funke *et al*., 2009; Yu *et al*., 2015) without a compensatory increase in expression. Before editing the EPSPS allele to produce high levels of glyphosate tolerance in cassava, it was therefore necessary to assess herbicide tolerance and fitness of plants with EPSPS variants expressed from the native cassava EPSPS promoter versus plants with the same variants expressed from a strong constitutive promoter.

## Results

**Figure 1:**
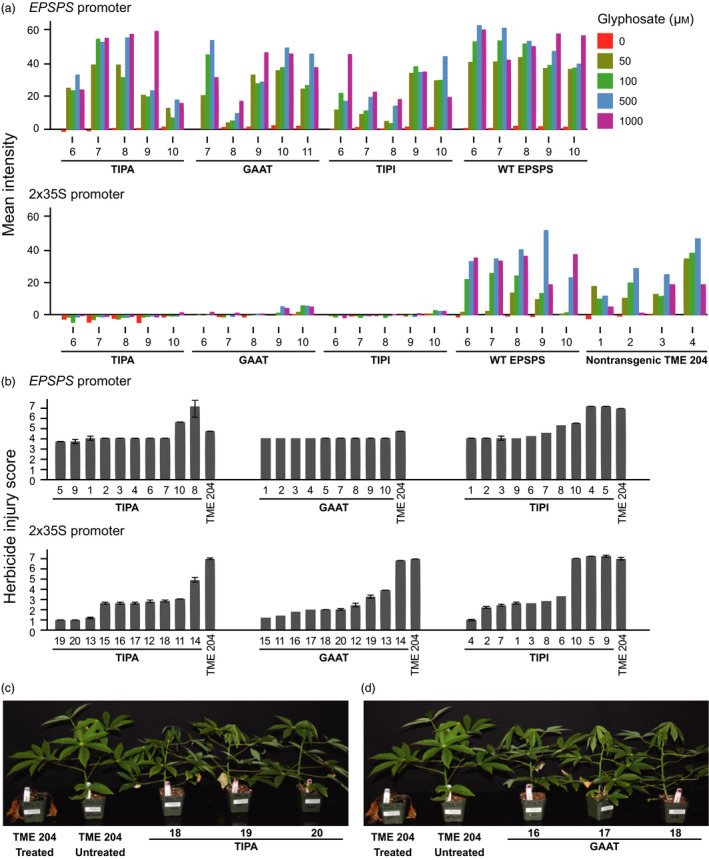
Glyphosate tolerance of cassava plants transformed with various EPSPS expression cassettes. (a) Quantification of shikimate accumulation in a colorimetric assay for the effect of glyphosate on EPSPS function in leaf discs derived from independent transformations with each of the *EPSPS* gene models. RGB images were converted to LAB space and average intensity of the ‘A’ channel was quantified for each sample. (b) Bar graphs indicating herbicide injury score of plants derived from independent transformation events of each gene model 23 days after application of 50 mg active ingredient (AI) of glyphosate isopropylamine salt to each plant. Impact of herbicide application was assessed three times per week on a scale of 1–7 for damage to the shoots where 1 = no damage to 7 = plant death. (c, d) Photographs showing representative phenotypes of plants derived from three independent transformations of the gene models expressing TIPA (c) and GAAT (d) mutant EPSPS enzymes from the 2x35S promoter 21 days after application of 50 mg AI of glyphosate isopropylamine salt to each plant. For all figures, numbers below bar graphs and photographs indicate the identity of independent transgenic events.

Two functional EPSPS genes exist in cassava, Manes.05G046900 and Manes.01G266800 (Bredeson *et al*., 2016). Of these, Manes.05G046900 was chosen for this study because it is expressed more strongly in 11 different tissues, including 25‐fold greater expression in the shoot apical meristem (Wilson *et al*., 2017). We generated a series of T‐DNAs with expression cassettes for three variants of EPSPS. These were expressed from either the cassava EPSPS promoter (2.2 kb of sequence upstream of the first codon), or by the tandemly repeated cauliflower mosaic virus 35S (2x35S) promoter, and are hereafter referred to as gene models (Figure S1). The 2x35S promoter exhibits strong, constitutive expression in cassava (Wilson *et al*., 2017). Each gene model was transformed into cassava cultivar TME 204 (Chauhan *et al*., 2015), and leaf discs of plants derived from transgenic events were tested for shikimate accumulation in the presence of glyphosate (Shaner *et al*., 2005). Leaf discs with enzymes expressed by the 2x35S promoter exhibited significantly higher EPSPS activity than discs with the same enzymes under control of the cassava EPSPS promoter, and double amino acid substitution variants were more tolerant to glyphosate than the wild‐type (WT) enzyme (Figures 1a and S2). Clonal replicates from 10 independent transformation events with each gene model were verified by endpoint PCR, propagated, established in the glasshouse and assessed for damage after foliar application of glyphosate isopropylamine salt at a rate of 50 mg AI per plant. As predicted by the shikimate accumulation assay, plants harbouring EPSPS enzymes expressed from the 2x35S promoter exhibited significantly less injury over the 23‐day observation period compared to plants with enzymes expressed from the native cassava EPSPS promoter (Figures 1b and S3). These observations indicate the importance of expression profile in glyphosate tolerance strategies based on plant EPSPS enzymes. The strongly expressed TIPA and G101A/A192T (GAAT) EPSPS variants supported more vigorous growth than the T102I/P106I (TIPI) enzyme (Figures 1c,d and S4), suggesting these alleles are also best suited to support plant fitness when the WT locus is permanently replaced by gene editing.

To assess which EPSPS expression cassettes enabled *in vitro* selection of modified cells, transformations with the gene model constructs were repeated, and plants regenerated on media containing 2.5–5 mm glyphosate. Of 30 recovered events, 29 were obtained with cassettes containing the 2x35S promoter driving expression of EPSPS variants (Table S1), demonstrating that strong expression is required for effective *in vitro* glyphosate tolerance. *In vitro* rooting assays, comparing the ability of micropropagated stem cuttings to form roots in media containing glyphosate (Figure S5), also indicated that the TIPA enzyme under control of the 2x35S promoter enabled growth of significantly more and longer roots compared to either the TIPA enzyme expressed from the native promoter or the WT enzyme under control of either promoter. Combined, these analyses show utility of the TIPA enzyme, when expressed under control of the strong constitutive 2x35S promoter, for providing tolerance to glyphosate in cassava.

**Figure 2:**
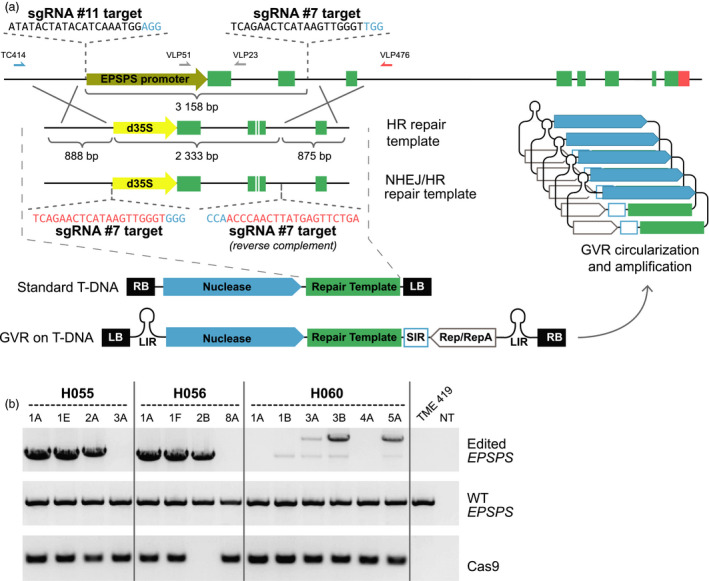
*EPSPS* editing strategies and molecular characterization of recovered plants. (a) Scaled map of the *EPSPS* locus, repair templates configured for exploitation of the HR pathway alone or for both the HR and NHEJ pathways, and the T‐DNA structures. Cas9 was expressed from the 2x35S promoter, while sgRNA #7 and sgRNA #11 were expressed from the *Arabidopsis* U6 and 7SL promoters, respectively. GVR = geminivirus replicon (b) PCR characterization of glyphosate‐resistant plants derived from the NHEJ or HR repair template on standard T‐DNA (H055), or the HR repair template on standard T‐DNA (H056) and on the GVR (H060). Gene targeting was assayed with the TC414/VLP476 primers under conditions that would not amplify the WT allele. Heterozygosity of the editing events was confirmed in all plants by detection of the WT allele with primers VLP51/VLP23. Identification of all amplicons was verified by Sanger sequencing (not shown).

With knowledge acquired from production and characterization of the EPSPS transgene models, we sought to generate plants with EPSPS alleles precisely edited for best tolerance to glyphosate. A strategy was developed to replace the endogenous EPSPS promoter and first two exons with a strong constitutive promoter and the TIPA amino acid substitutions. To achieve this, we identified CRISPR/Cas9 endonuclease (Jinek *et al*., 2012) targets in the promoter and second intron of the EPSPS locus that caused a 3.2‐kb deletion when co‐expressed in cassava protoplasts (Figure S6). Two repair templates were constructed to mediate correction of the disrupted locus by either homologous recombination (HR) or nonhomologous end‐joining (NHEJ; Figure 2a). Repair templates and expression cassettes for Cas9 and single‐guide RNAs (sgRNA) were combined in a standard cloning vector or incorporated in a vector as part of a geminivirus replicon (GVR) derived from the bean yellow dwarf virus (BeYDV) (Baltes *et al*., 2014). After verifying circularization of the BeYDV GVR in cassava callus cells (Figure S7), we transformed protoplasts and harvested genomic DNA after 48 h. Using a selective PCR amplification strategy, with one primer directed to the genomic EPSPS locus and one primer directed to the repair template, EPSPS gene editing was confirmed at the left and right junctions of events derived from both repair templates (Figure S8). Sanger sequencing of clones derived from editing events with the repair template configured for both NHEJ and HR repair indicated 14 of the 16 recovered junction sequences occurred by NHEJ‐mediated correction, while only two occurred by HR.

Having verified functionality of the gene editing components in transient systems, we transferred the editing reagents to T‐DNA vectors, transformed cassava cultivar TME 419 and regenerated plants under glyphosate selection. Of six independent glyphosate‐resistant plant lines recovered from repair templates on standard T‐DNAs, precise EPSPS editing was confirmed in four lines by PCR and DNA sequencing of both the edited allele (Figures 2b) and the left and right junctions (Figure S9). The presence of a precise right‐junction without left‐junction or complete allele amplicons suggested the remaining two glyphosate‐resistant lines – event #3 from the H055 vector and event #8 from the H056 vector – were derived from one‐sided events, in which extra vector sequence may have integrated at the left junction, preventing amplification over that region. This is consistent with the herbicide tolerance of these lines, because precise right‐junction events would be expected to produce a functional EPSPS cassette. Precise editing frequency as high as 0.13 events per cm^3^ settled cell volume (SCV) of embryogenic callus was achieved with the standard T‐DNA configured for HR‐mediated DSB repair – the most efficient editing strategy among those tested (Table 1). Compared to recovery of 16 transformation events per cm^3^ SCV obtained with the 2x35S‐driven TIPA gene model vector (Table S1), this represents an editing efficiency of about 1% of the frequency of random integration events recovered using the same selection system.

**Table 1:** *EPSPS* editing events recovered under 2.5–5 mm glyphosate selection

Vector	T‐DNA structure	Repair pathway	Transformations per cm^3^ cell volume	Cassava genotype	Glyphosate‐resistant plant lines	Precise HR events	Imprecise HR events
H055	Standard	HR & NHEJ	3/29.8	TME 419	3	2	1
H056	Standard	HR only	2/14.9	TME 419	3	2	1
H060	Replicon	HR only	3/32.2	TME 419	4	0	3
H004	2x35s TIPA gene model	Random integration	1/1.6	TME 204	26	N/A	N/A

Unexpectedly, several events were recovered that appeared to be the result of both NHEJ‐ and HR‐mediated repair. Sequences derived from the CRISPR target sites in the H055 NHEJ repair template were identified at the endogenous EPSPS gene that could only have been copied into this locus by HR. Interestingly, these sites carry mutations indicating that imprecise NHEJ disrupted the nuclease target sequence either before or after genomic incorporation of the repair template by HR (Figure S9). NHEJ may have resulted in excision of some HR‐mediated repair template integrations, which could explain the low recovery of events with this vector. That the two precise events in plants recovered from the H055 vector were derived from HR‐mediated repair contrasts with the dominant pattern of NHEJ repair by the same template in protoplasts.

**Figure 3:**
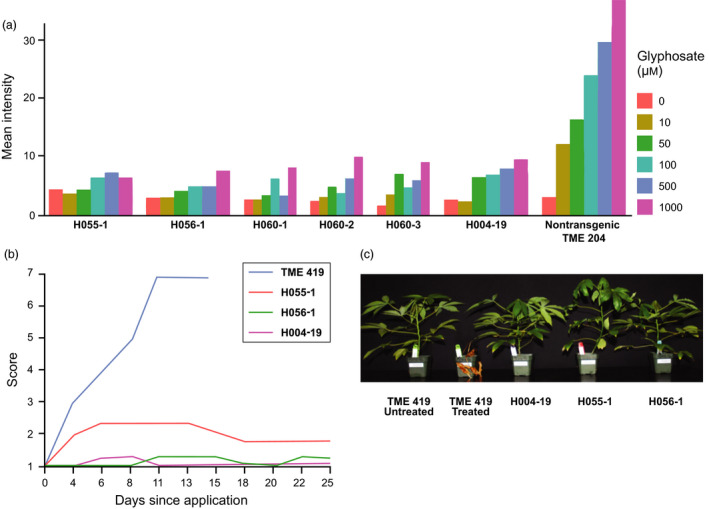
Glyphosate tolerance of plants with edited *EPSPS* alleles. (a) Quantification of shikimate accumulation. (b) Graph indicating average herbicide injury score of plants derived from *EPSPS* editing events with the H055 and H056 vectors. (c) Photograph showing representative phenotypes of plants derived from *EPSPS* editing events with the H055 and H056 vectors 21 days after application of 50 mg AI of glyphosate isopropylamine salt. Data shown in a, b and c were generated as described in Figure 1.

By increasing repair template abundance and inducing cell cycle phases that favour HR (Hanley‐Bowdoin *et al*., 2013), GVRs enhance homologous recombination efficiency in tobacco (Baltes *et al*., 2014), tomato (Čermák *et al*., 2015) and wheat (Gil‐Humanes *et al*., 2017). In this study, three of the four events derived with the BeYDV GVR appeared to contain precisely edited EPSPS; however, lines #3 and #5 also produced amplicons suggestive of imprecise EPSPS repair, with a large portion of the vector integrated at the target (data not shown). This, combined with the presence of the WT allele, suggests a complex genotype, or possibly mosaicism, in these lines. Furthermore, the edited lines – #1 (Figure 3; H060‐1), #3 (Figure 3: H060‐2, H060‐3) and #5 (Figure S9) from construct H060 – exhibited reduced vigour, stunting and leaf chlorosis. This possibly indicates an unfavourable match of the BeYDV GVR with cassava: pleiotropic effects of the replicase protein in cassava cells, presence of the replicon per se or provocation of a plant defence response may have inhibited regeneration of edited cells. A replicon derived from a cassava mosaic virus species, such as African cassava mosaic virus, may prove more effective for GVR‐based editing in cassava. The range of efficiencies observed from our various editing strategies highlights the importance of empirical testing within each system to identify the most effective approach for a particular species and transformation methodology.

Selectable marker‐free gene editing studies in wheat (Zhang *et al*., 2016) reported high frequency recovery of plants with targeted mutagenesis that did not contain detectable transgenic DNA. Our glyphosate selection strategy required high‐fidelity EPSPS editing to achieve herbicide tolerance, an outcome that could occur with or without concomitant integration of nuclease‐encoding T‐DNA sequences. A PCR screen for the presence of Cas9 indicated event #2, derived from the H056 vector, lacked detactable integration of the T‐DNA (Figure 2), which may suggest transgene‐free gene editing. Multiplexed gene editing may be achieved by selecting cells that have been edited for herbicide tolerance and then subsequently screening them for precise gene editing at a nonselectable locus.

To characterize functionality of the 2.3‐kb 2x35S TIPA sequence replacement and resulting glyphosate tolerance in regenerated plants, we assessed performance of one event derived from each of the standard T‐DNA vectors using the shikimate leaf disc assay (Figures 3a and S10) and foliar herbicide application (Figure 3b,c). The edited plants exhibited tolerance equivalent to the high‐performing plant line with the 2x35S‐driven TIPA gene model. This demonstrates the suitability of an EPSPS gene editing strategy to precisely introduce robust glyphosate tolerance in plants. As a new breeding tool for creating targeted genetic variation, gene editing enables efficient introduction of designed traits that are similar to what could occur in nature. With this in mind, our next objective is to introduce into the EPSPS allele a TIPA enzyme under control of a cassava strong constitutive promoter (Wilson *et al*., 2017) to generate a robust glyphosate tolerance trait without genomic integration of transgenic sequences.

We have applied precise gene editing to produce glyphosate tolerance in cassava at levels similar to conventional transgenic approaches. We found that direct selection for the EPSPS edit with repair template delivery on a standard T‐DNA configured to use only the HR repair pathway resulted in the highest recovery of edited events. One event may be lacking integration of the genome editing tool was indicated by PCR. In addition to the potential value of these specific lines for efficient weed control in cassava production, we anticipate this study will be used as a guide for the application of biotechnology to further improvements in cassava, as well as for the development of robust gene‐edited glyphosate traits in other crop species.

## Methods

### Plasmid assembly

Sequences used to build the gene model cassettes and repair templates were amplified from the Manes.05G046900 locus of cassava cultivar TME 7 genomic DNA with Q5 DNA polymerase (New England Biolabs). The Cas9 protein was previously described (Fauser *et al*., 2014) and was expressed from a double 35s promoter. sgRNAs (Jinek *et al*., 2012) #7 and #11 were synthesized (Integrated DNA Technologies, CA, USA) and cloned by Golden Gate Assembly (Engler *et al*., 2009) under control of the *Arabidopsis thaliana* U6 (Li *et al*., 2013) and 7SL (Baltes *et al*., 2015) promoters, respectively. Vectors were constructed primarily by Gibson Assembly (Gibson *et al*., 2009) from PCR amplicon and plasmid components. The BeYDV GVR was described previously (Baltes *et al*., 2014; Čermák *et al*., 2015). Agrobacterium tumefacien strain LBA4404 harbouring pCAMBIA2300‐based binary vectors (GenBank: AF234315) was used for the transformation of cassava.

### Cassava transformation

Transgenic and the gene‐edited events were generated in cassava cultivars TME 204 and TME 419, respectively, as described by Chauhan *et al*. (2015) with some modifications. For selection of transformed and gene‐edited events, two strategies were applied. In the first, friable embryogenic callus (FEC) inoculated with Agrobacterium containing H001, H002, H003, H004, H009, H010, H013, H014, H080 and H081 were selected on Gresshoff and Doy (Gresshoff and Doy 1974) basal medium supplemented with 2% w/v sucrose and 50 μm picloram (GD2 50P) supplemented with 27.5 μM paromomycin followed by subculture to media containing 45 μM paramomycin for embryo regeneration stages (Chauhan *et al*., 2015). For the second selection method, FEC was inoculated with Agrobacterium containing constructs H001, H002, H003, H004, H009, H010, H013, H014, H055, H056 and H060, and cultured on GD2 50P supplemented with 2.5 mm filter‐sterilized glyphosate [N‐(phosphonomethyl) glycine] (Sigma‐Aldrich, St Louis, MO). After 2 weeks, tissues were subcultured onto embryo regeneration stages (Chauhan *et al*., 2015) containing 5 mm glyphosate. Germination of mature somatic embryos took place on MS medium containing 2% w/v sucrose and 2 μm benzyl‐amino purine (BAP) without glyphosate. Regenerated plants were clonally micropropagated and established in soil as described previously (Taylor *et al*., 2012).

### Shikimate assays and quantification

Leaf discs 3 mm in diameter were excised from *in vitro* and glasshouse‐grown plants using a Mitex disposable biopsy punch with plunger (33‐32‐P/25) and silicone rubber stopper. Three leaf discs per plant were placed in a 48‐well plates and incubated in 200 μL of 10 mm ammonium phosphate (pH 4.4), 0.1% (v/v) Tween‐20 containing 0, 50, 100, 500 or 1000 μm glyphosate [N‐(phosphonomethyl) glycine] (Sigma‐Aldrich) and incubated at 24 °C under 200 μmol m^−2^s^−1^ light for 48 h. After incubation, the glyphosate solution was removed and 100 μL of 80% ethanol added to each well. The plates were placed in a −80 °C freezer for 30 min until the solution froze and then removed and thawed at room temperature for 10 min. The freeze–thaw process was performed three times and then 200 μL 1× shikimate assay solution added to each well. Plates were photographed 30 min later after the colour had fully developed. The 1× shikimate assay solution (20 mL) consisted of 2.5 mL 200 mm borate buffer (pH 9.0), 0.5 mL 18 mm nicotinamide adenine dinucleotide phosphate+, 0.25 mL 200 mm magnesium chloride, 1 mL 6 mm indonitrotetrazolium chloride, 0.1 mL diaphorase (100 U/mL), 5.3 mL of milli Q water and 0.001 mL of shikimate dehydrogenase enzyme. A shikimate standard curve was performed by addition of known amounts of shikimic acid to the empty wells. All the chemicals used for this assay were obtained from Sigma‐Aldrich.

The shikimic acid quantification (intensity of pink colour) was performed using ImageJ. RGB images were converted to LAB stack, and the A channel was selected as best able to differentiate the pink colour. Within each well, a region of interest (ROI) was selected avoiding the leaf discs as much as possible. ROIs were selected manually in ImageJ and were consistent in size across each image. Within each ROI, average intensity was measured using ImageJ. These intensities were graphed using R.

### *In vitro* rooting assays

Approximately 1.0‐ to 1.5‐cm‐long shoots were excised from 6‐week‐old micropropagated mother plants of TME 204 (Chauhan *et al*., 2015), events obtained from H001, H002, H003, H004, H080 and H081, and subcultured onto MS medium supplemented with 2% w/v sucrose solidified with 0.22% w/v phytagel. Filter‐sterilized glyphosate [N‐(phosphonomethyl) glycine, Sigma‐Aldrich] at 0.05 mm was added after autoclaving. Cultures were incubated under 16‐h photoperiod (75 μmol m^−2^s^−1^ irradiance) at 28 ± 1 °C. Root formation was assessed per shoot explant every week for 3 weeks.

### Foliar glyphosate applications and scoring

Transgenic and gene‐edited plants were established in the glasshouse (Taylor *et al*., 2012) and grown under 100% humidity on a mist bench for 7 days. This was followed by transfer to the bench under domes at 26 °C/25 °C (day/night) with 60%–90% relative humidity and a growth for a further 2 weeks before moving to the open bench and maintenance 32 °C/27 °C (day/night) and 70%–95% relative humidity growth (Taylor *et al*., 2012; Yadav *et al*., 2011). Glyphosate solution was prepared by mixing 10 ml glyphosate 5.4 (Keystone Pest Solutions, LLC, US) with 390 μL surface (90% Non‐ionic Surfactant, Keystone Pest Solutions, LLC, USA) and 2.61 mL water. Fifty microlitres of glyphosate solution (containing 50 mg active ingredient) was applied dropwise to the fourth leaf below the apical meristem when plants were approximately 4 weeks old or 7 cm tall. Damage to plants due to glyphosate application was assessed three times weekly on a scale of 1–7 where 1 =  no damage, 2 =  chlorotic youngest leaves, 3 =  chlorotic youngest leaves plus damage to the meristem, 4 =  death of meristem and chlorotic leaves, 5 =  death of meristem, chlorotic leaves, visible but minor stem necrosis, 6 =  death of meristem, chlorotic and dead leaves, over 50% stem necrosis and 7 =  complete plant death A total of six plants were treated with glyphosate per plant line, one plant was treated with surfactant only, and one plant was maintained as an untreated control.

### Protoplast transient editing assays

A protoplast isolation and transformation protocol (Zhang *et al*., 2012) was adapted for cassava with major alterations as follows: leaves of 6 weeks old *in vitro* cassava plants of cultivar 60444 or TME 204 were sliced and incubated for 14 h in enzyme solution (1.0% cellulase R10, 0.25% macerozyme R10, 0.45 m mannitol, 20 mm MES, 1× Murashige and Skoog salts and 0.1% bovine serum albumin, pH 5.7–5.8) while shaking at 20 rpm. Cells were filtered through a 40‐μm strainer, transferred directly to the top of the sucrose solution and centrifuged without a deceleration brake. Cells were washed twice before quantification and diluted to 10^6^ cells mL^‐1^ in MMG solution (0.4 m mannitol, 15 mm MgCl_2_ and 4 mm MES, pH 5.7). Transformation was performed by mixing 200 μL of cells with 40 μL DNA before adding 240 μL polyethylene glycol transformation buffer (40% PEG, 0.2 m mannitol, 100 mm CaCl_2_) and incubating for 20 min. All steps were performed with W5 solution (2 mm MES pH 5.7, 154 mm NaCl, 125 mm CaCl_2_, 5 mm KCl), except where a different solution is described. Cells were moved to six‐well plates and incubated for 48 h in W5 solution before extracting DNA for analysis. Extractions were performed with the DNeasy Plant Mini Kit (Qiagen Inc., Germantown, MD, USA) by pelleting the cells at max speed and starting at step seven of the manufacturer's protocol.

Selective PCRs to verify EPSPS gene editing in protoplasts were performed with Q5 polymerase according to the manufacturer's protocol.

## Additional information

All EPSPS amino acid coordinates described in this paper are conventionally numbered according to their position in mature plant enzymes as in Sammons and Gaines (Sammons and Gaines, 2014). Supplementary information is available online. Correspondence and requests for materials should be addressed to NJT.

## Author contributions

NJT, DFV, RB, AWH and RDC designed the experiments. AWH, TC and CGS designed and constructed the gene model and gene editing plasmids and performed molecular analysis of the edited plants. RDC conducted experiments to develop the protocols for rooting tests and cassava transformations using glyphosate selection. RDC and NJT supervised the cassava transformations, rooting tests, shikimate assays and foliar applications. AMM and AV performed molecular analysis of plants and Agrobacterium strains. AB developed the shikimate assay and whole plant glyphosate methods. RB performed supporting bioinformatics and data analyses. AWH, NJT and DFV wrote the manuscript. All authors read and approved the final manuscript.

## Conflict of interest

The authors have no conflict of interest to declare. AWH, RDC, RB, DFV and NJT are inventors on a patent application (PCT/US2017/013208) for the technology described in this work.

## Data availability

All data generated or analysed during this study are included in this published article and its supplementary information files.

## Supporting information


**Figure S1** Schematics showing the various *EPSPS* gene models introduced by *Agrobacterium* transformation to generate transgenic cassava plants for evaluating glyphosate tolerance.
**Figure S2** Colorimetric assay for the effect of glyphosate on EPSPS function in leaf discs derived from independent transformations with each of the *EPSPS* gene models.
**Figure S3** Comparative glyphosate tolerance over time of plants with the *EPSPS* gene models.
**Figure S4** Phenotypes of plants with each of the *EPSPS* gene models 21 days after application of 50 mg AI of glyphosate isopropylamine salt to each plant.
**Figure S5** *In vitro* glyphosate tolerance of cassava plants transformed with various *EPSPS* gene models.
**Figure S6** Validation of sgRNA activity at the cassava *EPSPS* locus.
**Figure S7** Verification of circularization in cassava cells by the GVR derived from the bean yellow dwarf virus.
**Figure S8** Confirmation of *EPSPS* editing in cassava protoplasts.
**Figure S9** Junction‐PCR analysis of recovered plants.

**Figure S10** Colorimetric assay for the effect of glyphosate on EPSPS function in leaf discs derived from independent *EPSPS* editing events with the H055 and H056 vectors, as indicated.

**Table S1** Transformation event recovery with various gene model vectors.
