# Supplementary material for: Allele exchange at the EPSPS locus confers glyphosate tolerance in cassava
Source: Plant Biotechnol J. 2018 Jan 22;16(7):1275–82. doi: 10.1111/pbi.12868 (PMC5999311; doi:10.1111/pbi.12868)
Supplement: Supplementary file 1 — Figure S1 Schematics showing the various EPSPS gene models introduced by Agrobacterium transformation to generate transgenic cassava plants for evaluating glyphosate tolerance. Figure S2 Colorimetric assay for the effect of glyphosate on EPSPS function in leaf discs derived from independent transformations with each of the EPSPS gene models. Figure S3 Comparative glyphosate tolerance over time of plants with the EPSPS gene models. Figure S4 Phenotypes of plants with each of the EPSPS gene models 21 days after application of 50 mg AI of glyphosate isopropylamine salt to each plant. Figure S5 In vitro glyphosate tolerance of cassava plants transformed with various EPSPS gene models. Figure S6 Validation of sgRNA activity at the cassava EPSPS locus. Figure S7 Verification of circularization in cassava cells by the GVR derived from the bean yellow dwarf virus. Figure S8 Confirmation of EPSPS editing in cassava protoplasts. Figure S9 Junction‐PCR analysis of recovered plants. [file PBI-16-1275-s003.pdf]

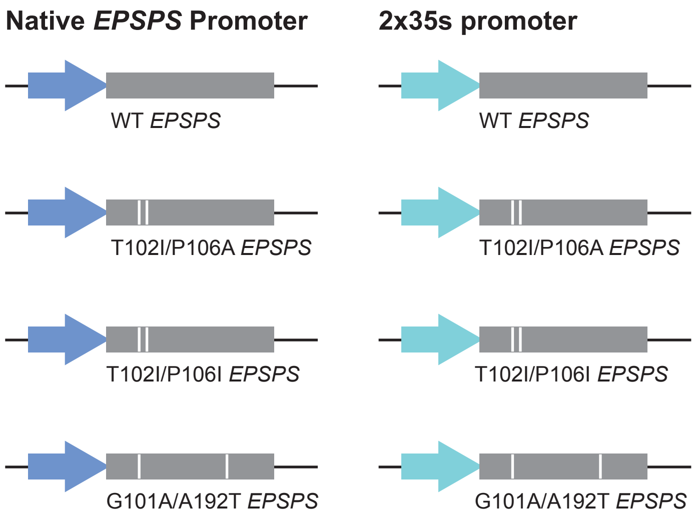

Supplemental Figure 1. Schematics showing the various *EPSPS* gene models introduced by Agrobacterium transformation to generate transgenic cassava plants for evaluating glyphosate resistance. Block arrows indicate the promoters, and amino acid substitutions are marked with white vertical bars. Not drawn to scale. Introns are not indicated.

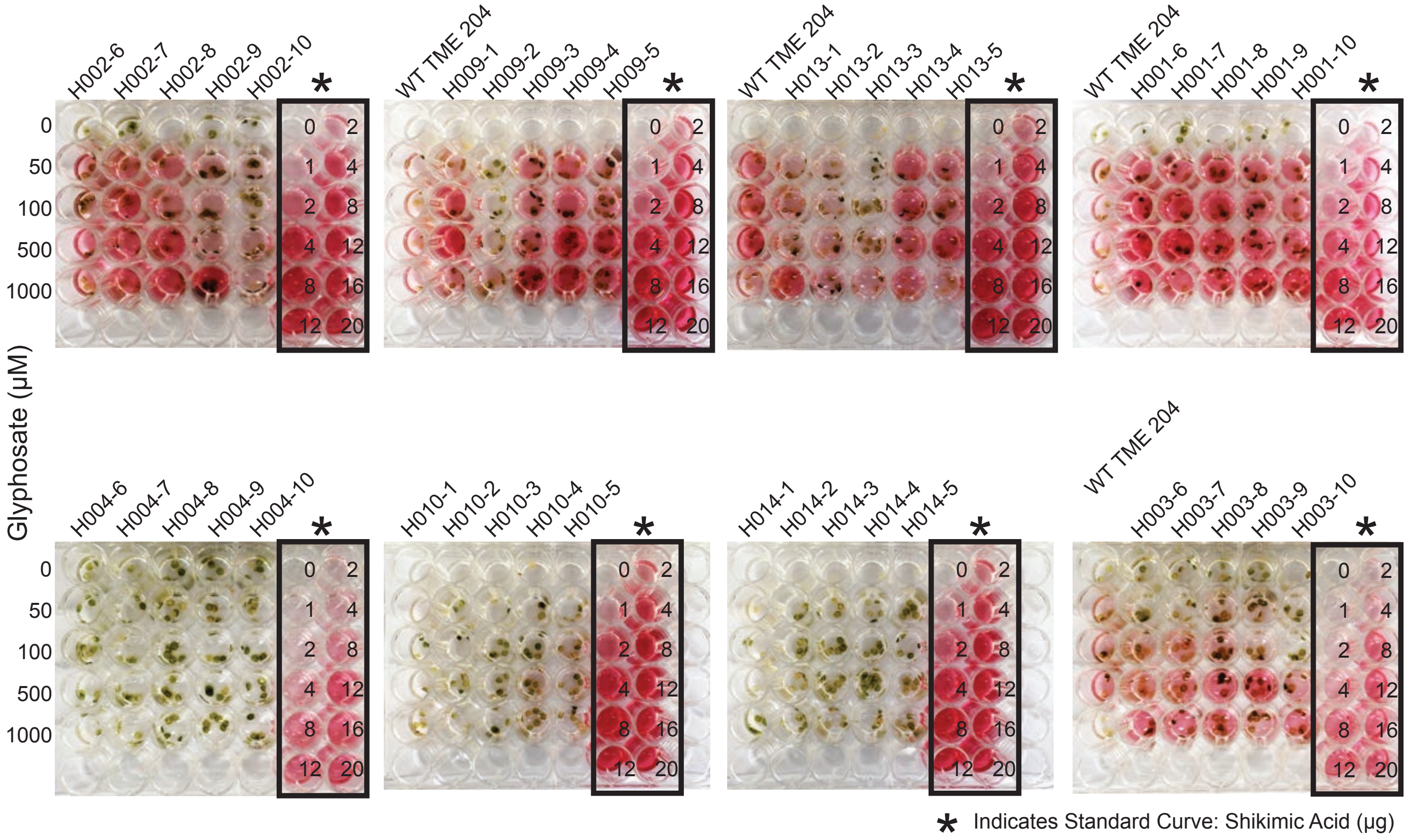

Supplemental Figure 2. Colorimetric assay for the effect of glyphosate on EPSPS function in leaf discs derived from independent transformations with each of the *EPSPS* gene models, as indicated. Pink color indicates the accumulation of shikimate due to inhibition of *EPSPS* activity. Coloring of each well was quantified and normalized to the appropriate standard curve to generate the graph shown in Figure 1a.

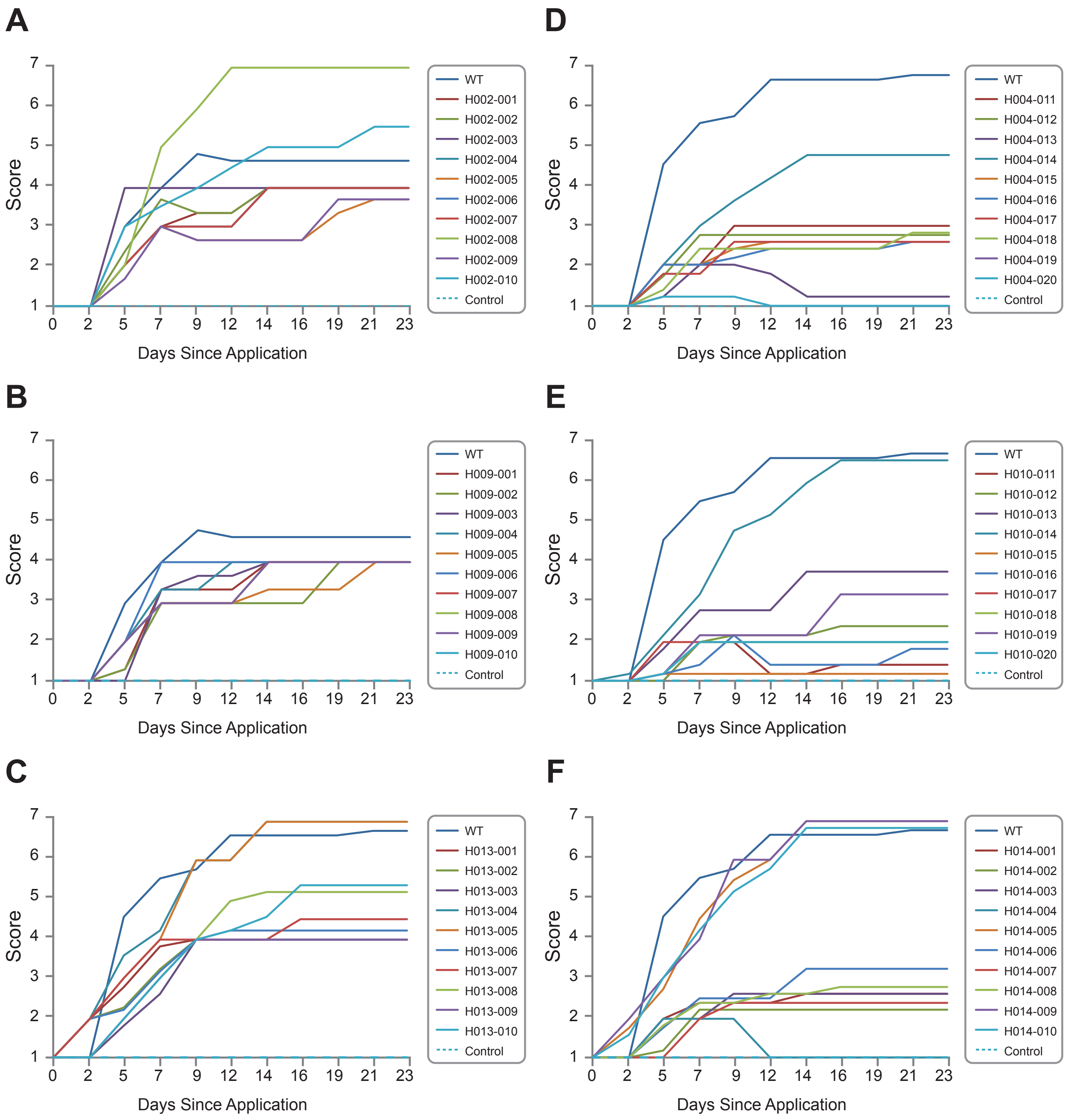

Supplemental Figure 3. Comparative glyphosate tolerance over time of plants with the *EPSPS* gene models. Injury scores of plants from independent lines with the EPSPS promoter driving **A)** TIPA, **B)** GAAT, or **C)** TIPI enzymes; and plants with the double 35s promoter driving **D)** TIPA, **E)** GAAT, or **F)** TIPI enzymes after application of 50 mg of glyphosate isopropylamine salt. Impact of herbicide application was assessed three times weekly on a scale of 1-7 for damage to the aerial portions where 1 = no damage to 7 = plant death. Average scores from the 23 day time point were graphed in Figure 1b.

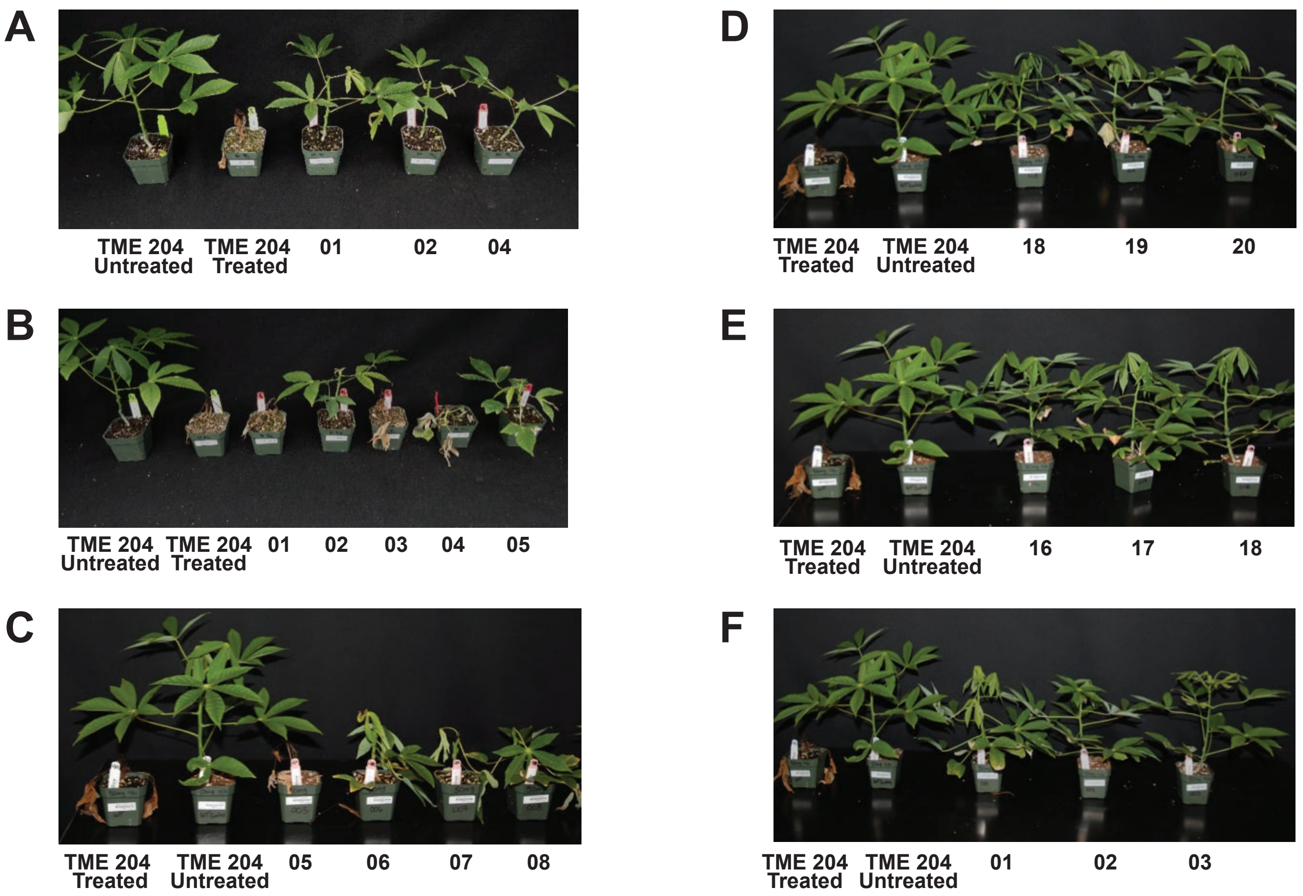

Supplemental Figure 4. Phenotypes of plants with each of the *EPSPS* gene models 21 days after application of 50 mg of glyphosate isopropylamine salt. Representative independent lines of plants with the *EPSPS* promoter driving **A)** TIPA, **B)** GAAT, or **C)** TIPI enzymes; and plants with the double 35s promoter driving **D)** TIPA, **E)** GAAT, or **F)** TIPI enzymes enzymes were selected.

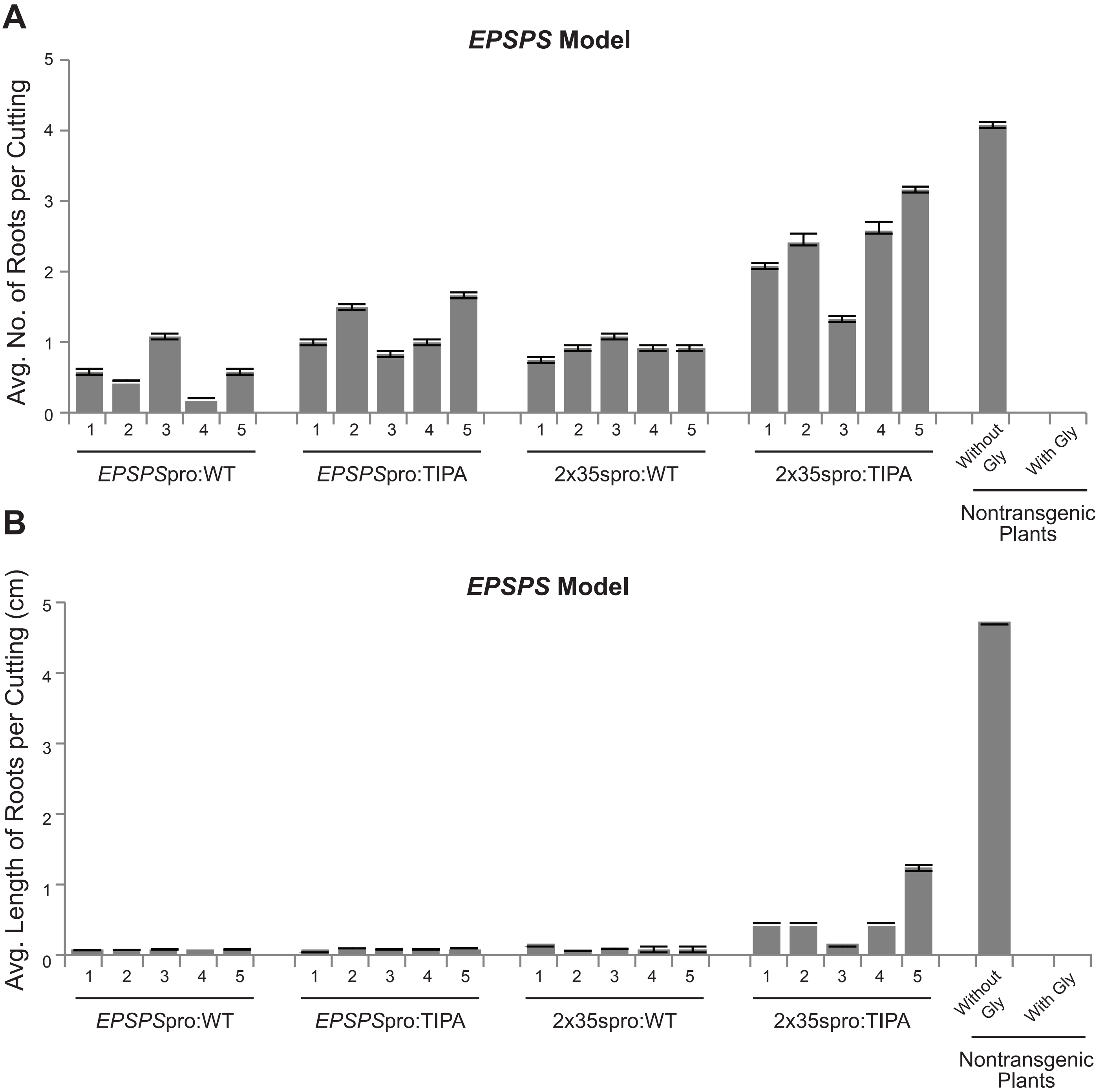

Supplemental Figure 5. Glyphosate tolerance of cassava plants transformed with various *EPSPS* gene models. a, b, In vitro growth assays indicating the number **A)** and length **B)** of roots formed in the presence of glyphosate by stem cuttings derived from five independent events of *EPSPS* cassettes expressing the WT or TIPA enzymes from either the *EPSPS* promoter or the double 35s promoter, as indicated.

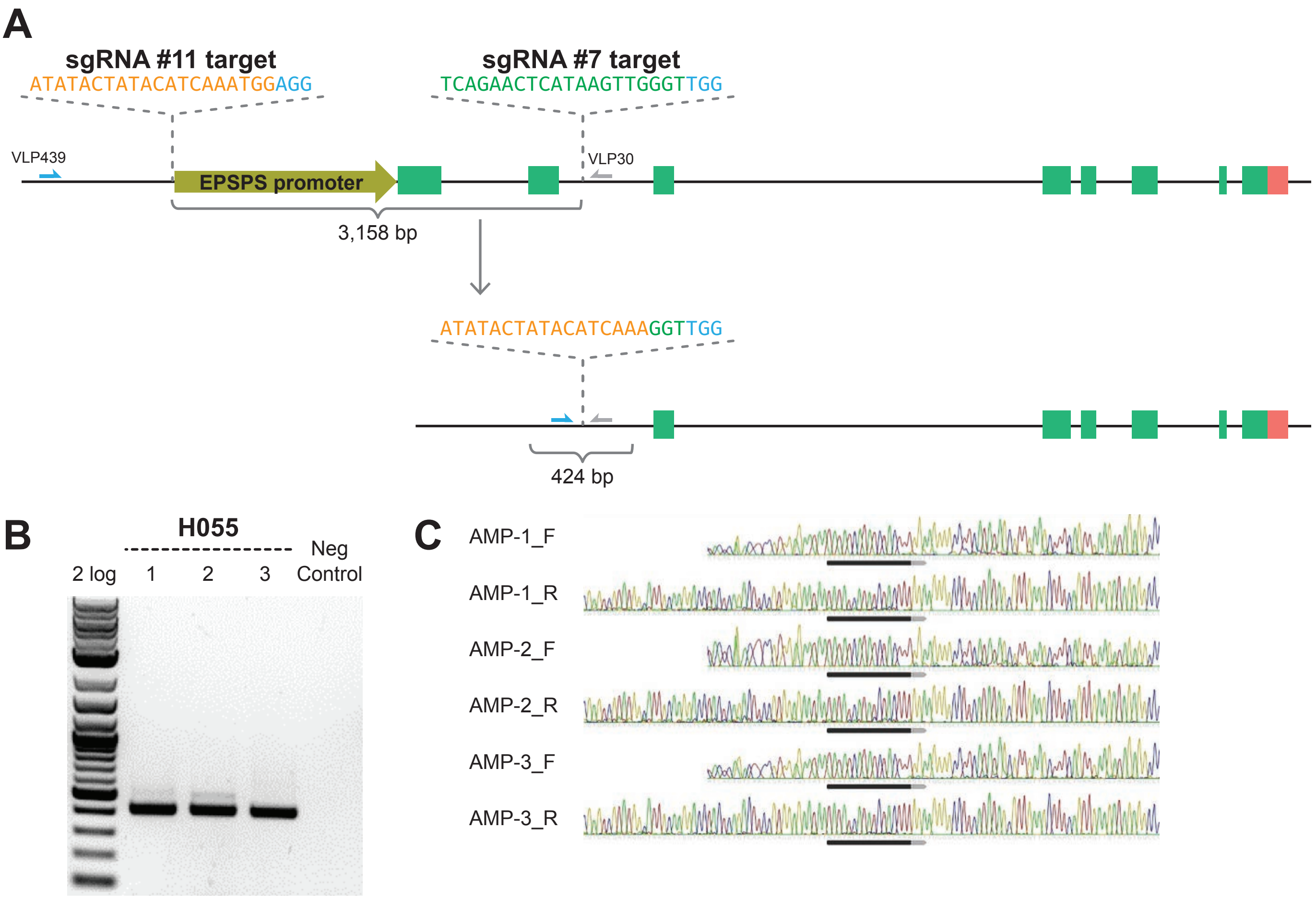

Supplementary Figure 6. Validation of sgRNA activity at the cassava *EPSPS* locus. A) Scaled map of *EPSPS* showing cutting sites of sgRNA #7 and sgRNA #11, and approximate binding locations of the primers used to detect presence of the deletion caused by cutting at both nuclease targets followed by repair with loss of the intervening sequence. **B)** Agarose gel showing the presence of the expected 424 bp amplicon of the VLP439/VLP30 primer pair derived only from each of three protoplast samples treated with varying amounts of a plasmid expressing Cas9, sgRNA #7, and #11, but not from an untreated control. PCR conditions selectively amplified deletion events and were not suitable for amplification of the WT allele. Plasmid quantities transformed in each sample were: 1 = 30 µg, 2 = 28.8 µg, 3 = 7.5 µg, neg control = 25.8 ug. **C)** Sequence verification of the three deletion amplicons from (b) showing that the majority of each amplicon population represents clean deletion-ligation events without any addition or loss of nucleotides from the outer chromosomal breaks. Each amplicon was sequenced in both directions toward the site of ligation. Portions of the fused sgRNA #11 and sgRNA #7 target sites are indicated on the sequencing traces by dark and light gray annotations, respectively.

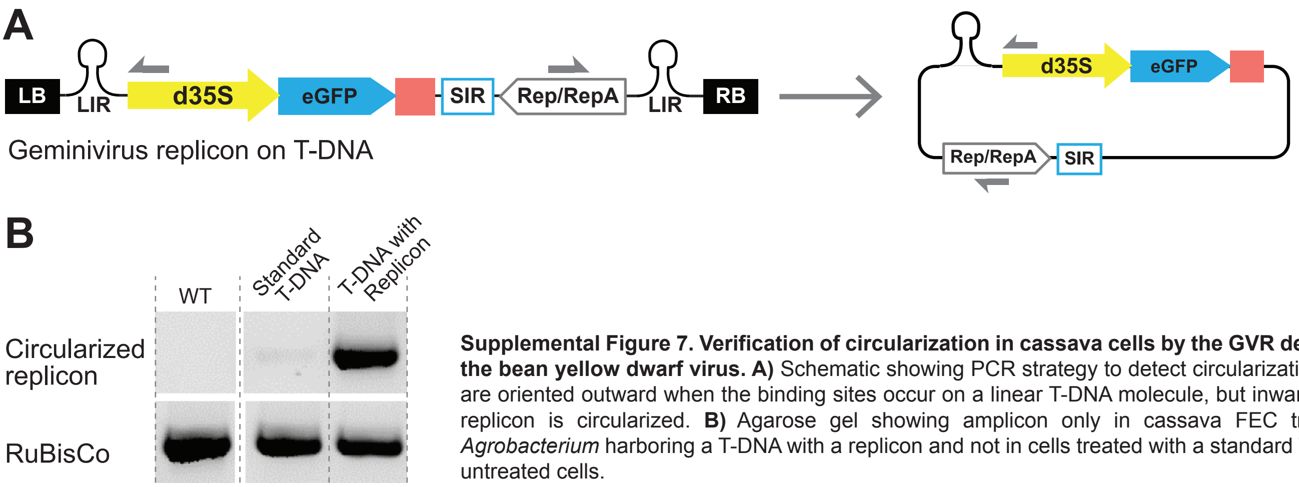

Supplemental Figure 7. Verification of circularization in cassava cells by the GVR derived from the bean yellow dwarf virus. A) Schematic showing PCR strategy to detect circularization. Primers are oriented outward when the binding sites occur on a linear T-DNA molecule, but inward when the replicon is circularized. **B)** Agarose gel showing amplicon only in cassava FEC treated with *Agrobacterium* harboring a T-DNA with a replicon and not in cells treated with a standard T-DNA or in untreated cells.

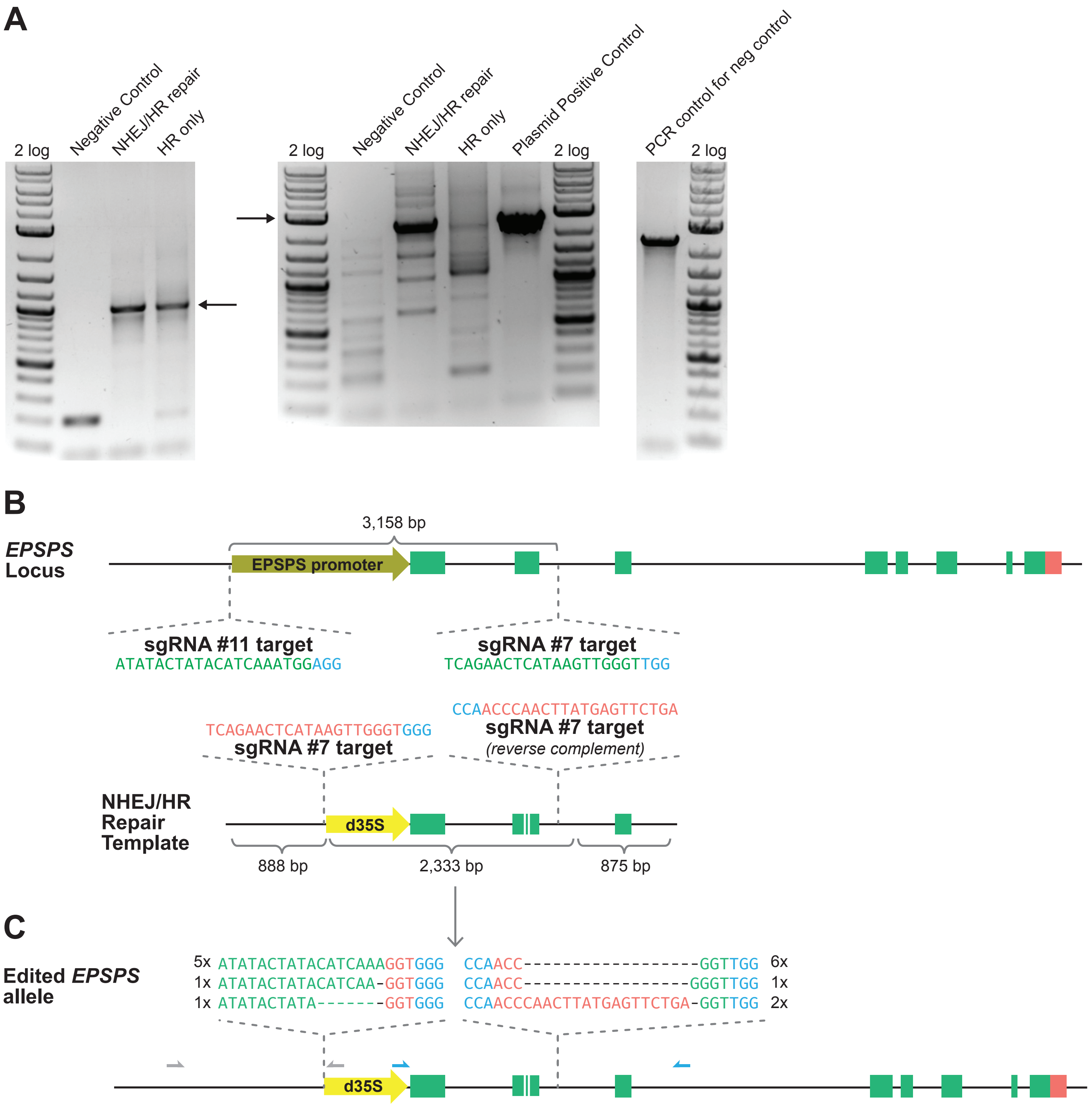

Supplementary Figure 8. Confirmation of *EPSPS* editing in cassava protoplasts. A) Amplicons derived from protoplasts transformed with vectors expressing CRISPR/Cas9 endonucleases and carrying repair templates as described in Figure 2. Arrows indicate bands of the expected size for editing at the left- and right-junctions, as indicated. **B)** Scaled map of the *EPSPS* locus and repair template configured for NHEJ and HR, as shown in Figure 2. **C)** Scaled map of the edited *EPSPS* allele showing sequences and frequencies of clones derived from the junction PCR amplicons from cells treated with the repair template configured for NHEJ and HR. All left- and seven of the right-junction sequences indicate NHEJ-mediated sequence replacements, while the remaining two right-junction events represent HR-mediated sequence replacements. The event-specific amplification strategy was similar to what is described in Figure 2.

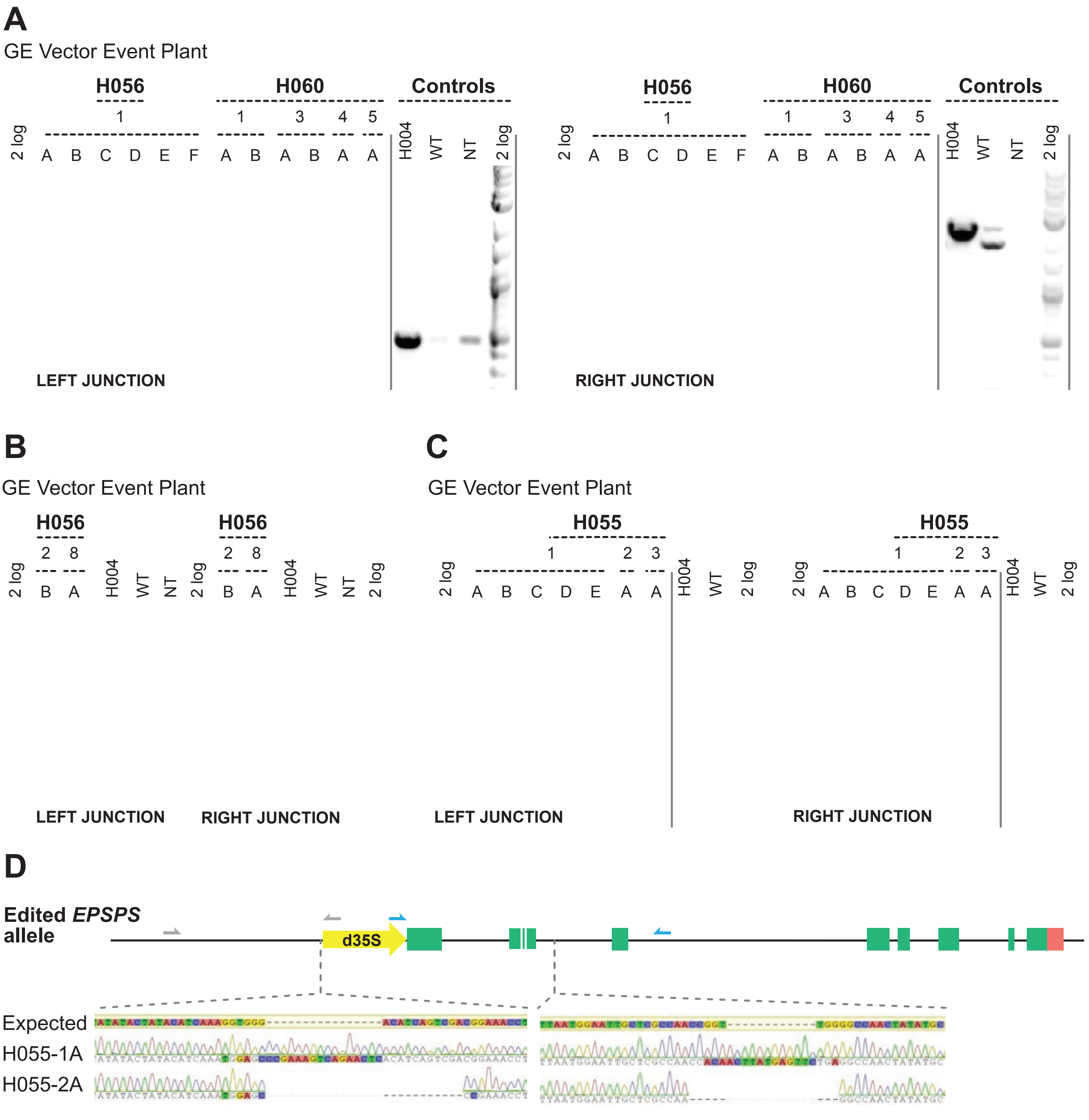

Supplementary Figure 9. Junction-PCR analysis of recovered plants. A-C) PCR-characterization of glyphosate resistant plants derived from the HR repair template on standard T-DNA (H056) and on the GVR (H060) **A, B)** and of glyphosate resistant plants derived from the NHEJ or HR repair template on standard T-DNA (H055) **C)** showing the presence of a left junction and right junction, by amplification with primers pairs TC414/TC415, and VLP498/VLP476, respectively. Events #3 and #8 derived with vectors H055 and H056, respectively, likely represent one-sided HR due to the presence of properly sized right junction but without specific amplification of the left junction. **D)** Scaled map of the edited *EPSPS* locus with amplicons sequences obtained from H055 events #1 and #2 at the location where NHEJ-mediated integration would be expected to occur. Expected sequence indicates a clean NHEJ repair event with no loss or addition of nucleotides from the cleaved repair template or chromosomal ends. The presence of vector sequence in both left junctions and the H055 event #1 right junction indicates at least these junctions were integrated by HR.
