# Supplementary material for: Allele exchange at the EPSPS locus confers glyphosate tolerance in cassava
Source: Plant Biotechnol J. 2018 Jan 22;16(7):1275–82. doi: 10.1111/pbi.12868 (PMC5999311; doi:10.1111/pbi.12868)
Supplement: Supplementary file 2 — Figure S10 Colorimetric assay for the effect of glyphosate on EPSPS function in leaf discs derived from independent EPSPS editing events with the H055 and H056 vectors, as indicated. [file PBI-16-1275-s002.pdf]

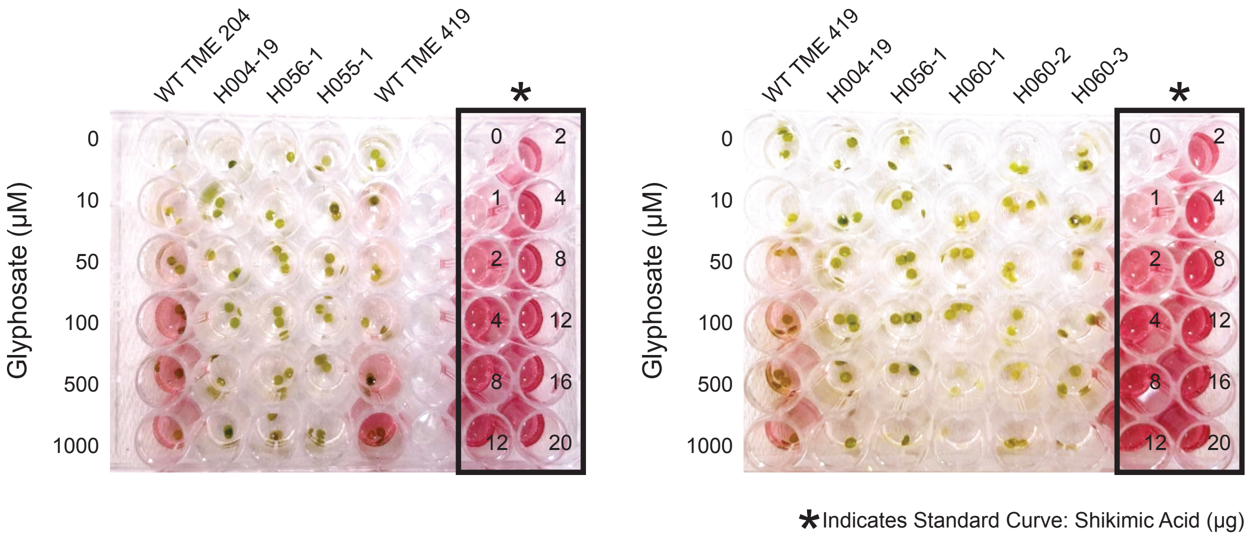

Supplemental Figure 10. Colorimetric assay for the effect of glyphosate on *EPSPS* function in leaf discs derived from independent *EPSPS* editing events with the H055 and H056 vectors, as indicated. Assays were performed as described for Figure S2. Coloring of each well was quantified and normalized to the appropriate standard curve to generate the graph shown in Figure 3a.
