# Supplementary material for: Allele exchange at the EPSPS locus confers glyphosate tolerance in cassava
Source: Plant Biotechnol J. 2018 Jan 22;16(7):1275–82. doi: 10.1111/pbi.12868 (PMC5999311; doi:10.1111/pbi.12868)
Supplement: Supplementary file 3 — Table S1 Transformation event recovery with various gene model vectors. [file PBI-16-1275-s001.pdf]

Supplemental Table 1. Transformation event recovery with various gene model vectors

T-DNA Vector	Gene model configuration		Events recovered per cm^3 settled cell volume	
	Promoter	*EPSPS* variant	Paromomycin selection[b]	Glyphosate selection[b]
H001	Native *EPSPS*[a]	WT	10	0
**H003**	**2xCaMV35s**	**WT**	**28**	**0**
H002	Native *EPSPS*[a]	T102I/P106A	14	0
**H004**	**2xCaMV35s**	**T102I/P106A**	**17**	**16**
H009	Native *EPSPS*[a]	G101A/A192T	6	1
**H010**	**2xCaMV35s**	**G101A/A192T**	**14**	**11**
H013	Native *EPSPS*[a]	T102I/P106I	4	0
**H014**	**2xCaMV35s**	**T102I/P106I**	**5**	**2**

[a] The *EPSPS* promoter sequence was isolated from cultivar TME 7

[b] Selective agents were applied independently
